# Evaluation of Malware Classification Models for Heterogeneous Data

**DOI:** 10.3390/s24010288

**Published:** 2024-01-03

**Authors:** Ho Bae

**Affiliations:** Department of Cyber Security, Ewha Womans University, Seoul 03760, Republic of Korea; hobae@ewha.ac.kr

**Keywords:** adversarial learning, IoT, deep learning, interpretability, XAI for CTI applications, XAI for cybersecurity data

## Abstract

Machine learning (ML) has found widespread application in various domains. Additionally, ML-based techniques have been employed to address security issues in technology, with numerous studies showcasing their potential and effectiveness in tackling security problems. Over the years, ML methods for identifying malicious software have been developed across various security domains. However, recent research has highlighted the susceptibility of ML models to small input perturbations, known as adversarial examples, which can significantly alter model predictions. While prior studies on adversarial examples primarily focused on ML models for image processing, they have progressively extended to other applications, including security. Interestingly, adversarial attacks have proven to be particularly effective in the realm of malware classification. This study aims to explore the transparency of malware classification and develop an explanation method for malware classifiers. The challenge at hand is more complex than those associated with explainable AI for homogeneous data due to the intricate data structure of malware compared to traditional image datasets. The research revealed that existing explanations fall short in interpreting heterogeneous data. Our employed methods demonstrated that current malware detectors, despite high classification accuracy, may provide a misleading sense of security and measuring classification accuracy is insufficient for validating detectors.

## 1. Introduction

Numerous empirical studies have demonstrated the significant potential and effectiveness of machine learning (ML) in addressing specific security issues (i.e., malware detection [1,2]). ML techniques for detecting malware have been developed over the years and applied in a variety of security domains, including the clustering of malware families [3,4], the identification of malicious downloads [5,6], the detection of account misuse networks [7,8], and the recognition of commonly exploited file formats such as Java archives [9], documents [10,11], and PDF malware [12,13,14,15]. Among these file types, PDF and portable executable (PE) files have gained considerable popularity in both cyber attacks and defense strategies. SonicWall [16] has reported a significant rise in new attacks related to PDF and PE files, with over 268,362 new attacks discovered in 2020, reflecting a substantial 74% year-over-year increase.

The inaugural PDF classification model, PDFrate-v1 [13], addressed the challenge of PDF document classification using ML techniques, leveraging both metadata and the content of PDF documents. This approach involved manually crafting 202 features to train a random forest (RF) model for detection. Subsequently, PDFrate-v2 [14] enhanced the performance by employing an ensemble training technique. Hidost [12,15] took a different approach involving the extraction of files into a structural map and utilizing it as a feature set for a training model. Support vector machines (SVMs) and RF have been employed as classification models with impressive detection performance. A recent study [17] introduced a verifiable robust classifier with subtree deletion and insertion properties, which enable the classifier to withstand attacks involving a bounded number of subtrees under the root node. To mitigate such attacks, Ref. [17] advocated for verifiably trained models that exhibit robustness against all possible bounded attacks.

Since the inception of research on adversarial examples (AEs) in machine learning (ML) [18,19,20,21], it has been known that slight perturbations to the input can substantially alter the prediction outcomes. Subsequently, the range of attacks has expanded to encompass the field of malware. This development poses challenges for ML model developers focused on malware [22], making it imperative to construct robust malware detectors or classifiers that can withstand AE attacks. A strategy commonly employed by many researchers and developers involves fortifying a model by training it against all conceivable AEs, leading to considerable efforts in developing malware detection systems that identify AEs that target existing ML models. From recent AE attacks [23,24,25], it can be seen that AE attacks have a stronger effect on poisonous malware-based detectors, with the accuracy of most high-performing models converging to zero with a false positive rate of under 0.1%.

In assessing ML classifiers for security applications, it is inadequate to rely solely on accuracy and false positive rates. Although a wide variety of metrics have been developed in ML theory, none comprehensively measure the robustness of classifiers against AEs. For a malware classifier to be practically effective, it must demonstrate security against various security measures. For example, a robustness property might require the classifier to correctly identify modified malware as malicious, even if benign features have been inserted into a PDF malware or vice versa. This emphasizes the importance of evaluating classifier security using alternative metrics to avoid a false sense of confidence in their performance. Our goal in this study was to understand the rationale for the effectiveness of the AEs on malware classifiers. We suspected that the performance of the existing classifiers is determined simply by the prediction accuracy, which typically provides a false sense of the model. To resolve this problem, it was necessary to view it from the perspective of model interpretation rather than relying on simple results.

Explainable AI (XAI) is an approach to solving the above-mentioned problem of false security based on prediction accuracy. XAI methods can be categorized as either interpretable models [26,27] or post hoc explanations [28,29,30]. The former utilize built-in architectures for interpretability to expose the components of input data that the model focuses on when making a decision. Several studies have proposed methods for interpreting a face recognition model by using interpretable models [31,32]. Later post hoc explanations have interpreted a trained deep neural network (DNN) by fitting explanations of how the model performs the classification.

Several XAI methods based on the post hoc approach have been proposed [26,27,28,29,30] on the basis of the post hoc approach. To interpret image models, such XAI models assign an importance score to each pixel of the input data utilizing built-in architectures to expose the parts of the input data that the model focuses on when making a decision. Later studies of XAI have interpreted trained DNNs by fitting explanations of how the models perform classification. However, several problems have made it difficult to explain the models using previous XAI methods as a result of (a) the complexity of deep learning models, (b) the existence of near-zero gradients for saturated predictions, and (c) the dependence of the sensitivity on the values of the inputs. Although several studies have attempted to tackle such problems [30,33], these methods cannot be applied to heterogeneous data because they are dependent on the homogeneous nature of the target input data such as image pixels.

For heterogeneous data, we use black-box explanation methods, which are able to explain any model [28,34,35,36], used to evaluate classifiers. The most commonly used post approach is the locally interpretable model-agnostic description (LIME), which operates the opaque models that are trained using text, tabular, and image datasets as a black-box.

**Figure 1:**
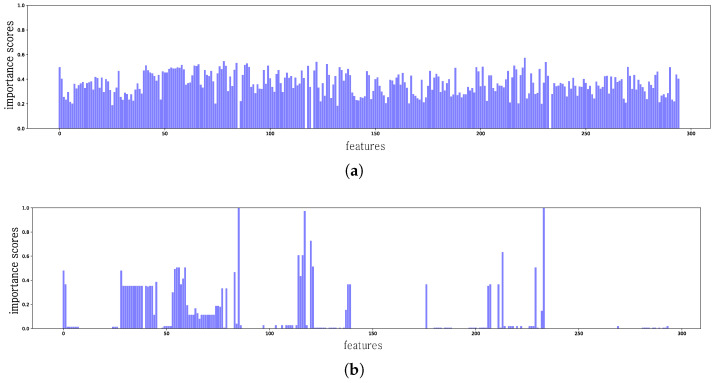
The sum of the importance scores of the 295 features in the PDF dataset using LIME [28]. We set the number of representative instances as eight, four instances from each class. (**a**) The sum of importance scores by using LIME. (**b**) Ground truth test attack distribution.

The framework [37] accounts for the predictions of datasets by capturing the local opaque model using the regional data distribution. In this process, a surrogate classifier is stuck to the opaque model in the surrounding small neighborhood and an interpretable classifier is developed based on a linear model trained with a given dataset to obtain high fidelity to the original model. XAI methods for the interpretation of the classification task generally use the logit gradient of the ground-truth label with respect to the input. If the original model function behaves linearly in the space of neighboring samples around the input datasets, the distribution of the input data can vary depending on the problem being solved, which might not be accounted for by a fixed sampling method. To obtain a global model explanation, LIME employs a submodular pick that uses the non-redundant coverage explanations of all features. However, current LIME approaches cannot distinguish importance features by the type of data, as shown in Figure 1a. In addition, Ref. [38] showed that the importance scores generated by LIME are not robust to changes in the input values, which means that the importance scores of an individual feature can be dependent on the sensitivity of the feature.

In this paper, we demonstrate that the current LIME approach, specifically the submodular pick, is not capable of interpreting heterogeneous data, as mentioned in [38]; thus, we propose a method to select the top-k importance features to robustly utilize methods for the explanation of heterogeneous data. We used Bayesian optimization to identify an optimal solution for gradient-based optimizers to carry out an ensemble approach to the voting importance measurement of each feature. Figure 1a,b show concrete examples of misleading explanations presented by the current LIME. Figure 1a shows a distribution that is completely unrelated to the original test distribution in Figure 1b. We validated our approach using representative PDF and PE classifiers and demonstrate that the current explanation approaches can provide false explanations and unreliable results in representing their models. The analysis conducted via our interpretation model revealed that the latest AE attacks are effective against current malware classifiers because the classifiers rely on specific features that are targeted by AE attackers to diminish their effectiveness.

## 2. Background

### 2.1. Types of Model Verification

Attack scenarios can be classified into two categories based on the varying levels of knowledge possessed by the attacker: white-box attacks and black-box attacks. The degree of information available to an attacker determines the classification of the attack scenario, with a higher level of obscurity indicating a black-box attack. Three types of information can be disclosed to an attacker: (1) the training dataset along with its labels; (2) the feature set and the feature extraction algorithm of the classifier, including the types of features extracted; (3) the knowledge of the classification function and its hyperparameters.

Verification types follow the same principle as attack scenarios. In the white-box approach, the verifier has full knowledge of the classifier. However, this assumption is not realistic in practice because the provers are required to open their model to the verifier. For this reason, a black-box approach such as LIME [28] (described in Section 2.3) is preferred to the white-box approach. A black-box approach only allows for an attacker with minimal knowledge of all three types of information. The attacker is only given certain details of the feature representation (e.g., the feature types), providing only a vague idea of the modification strategy.

### 2.2. Types of Malware Classifiers

ML techniques for malware detection have undergone extensive development over the years and have been extended to various security domains. In particular, classifiers for PDF and PE files are widely employed owing to their ability to facilitate large-scale infections and broaden the scope of targeted attacks. The predominant approach for malware classifiers involves static and dynamic analysis. Static analysis involves scanning binary byte streams, printable strings, n-grams, instructions, etc., whereas dynamic analysis executes individual software components in an isolated environment (virtual box) to collect runtime behavior logs. Notably, our interpretation approach is designed to function in both static and dynamic analysis settings. For static analysis, we utilized three representative PDF classifiers, covering diverse types of PDF attacks such as JavaScript- and ActionScript-based attacks and file-embedding attacks. To carry out dynamic analysis, we employed a state-of-the-art method that incorporates code obfuscation and addresses zero-day malware. This comprehensive approach allows for a robust interpretation of malware characteristics across both the static and dynamic analysis methodologies.

Many PDF classifiers make use of the Poppler PDF parser [39] in the initial stage of structure extraction. Poppler is employed to break PDF files down into structural multi-maps. In this structural extraction stage, the paths of objects within a PDF are extracted and utilized as features during the classification process. To address the presence of numerous semantically equivalent, yet syntactically different structures, a process called structural path consolidation (SPC) is performed. SPC involves the application of manually created rules to consolidate and standardize structural paths for more-effective and consistent classification. Hidost, one of the first representative PDF classifier models, is provided as open-source. Hidost is utilized in SVM [12] and RF [15]. SVM, a supervised learning model approach, establishes an optimal hyperplane for distinguishing between two labels; RF, a meta-estimator, integrates multiple decision trees to enhance classification accuracy. In the initial phase, Hidost employs the Poppler PDF parser to deconstruct files into structural multimaps during the structure-extraction process. The features for classification are derived from the structural paths of objects in the PDF. To address semantically equivalent, but syntactically different structures, Hidost consolidates structural paths based on predefined rules. For feature selection, a rule similar to the SL2013 method is applied, including the use of only those paths that occur in more than a specified number of files. As an open-source tool, Hidost trained its model using a randomly selected set of 10,000 files with a 1:1 ratio of malicious to benign samples. The PDF dataset comprises 407,037 benign and 32,567 malicious files.

PDFrate classifier is utilized in RF and ensemble models to improve prediction accuracy [14] by employing both metadata and PDF file content, including the author name, file size, and specific keywords as features. Its feature set, manually defined by the authors, comprises 202 features, with 135 publicly available in the Mimicus implementation of PDFrate, which are claimed to closely match the full feature set’s performance. PDFrate-v1 and -v2 differ from the original version primarily in adopting the deep learning (DL) model. PDFrate-v2 uses an ensemble method, introducing “Uncertain” in the classifier votes, with 25–50 considered uncertain (benign) and 50–75 uncertain (malicious). PDFrate-v2 effectively counters evasive attacks such as mimicry and reverse mimicry, demonstrating impressive performance against these challenges.

The verifiably robust PDF classifier [17] measures the distance between pairs of PDFs in terms of the number of subtrees and the depths of the respective PDF trees. Most attacks operate using the PDF object insertion and deletion mutation operations and optimize the search space based on the feedback from the classifiers. Ref. [17] attempted to make the search space less vulnerable to (insertion/deletion) operations and tested the effectiveness of their approach against all recent attacks, including mimicry [25], reverse mimicry [40], EvadeML [41], EvadeHC [42], and AE attacks [22]. Most early studies on dynamic analysis [43,44,45,46] considered only statistical information and ignored API arguments, preventing them from fully exploiting heterogeneous information. For instance, statistical information captures only spatial features such as the mean, variance, and entropy. To capture information on API arguments, Ref. [47] proposed a feature representation associating API calls with their corresponding arguments, using hashing-based approaches to extract the heterogeneous information and claimed that their method outperformed all baselines.

### 2.3. Overview of LIME

In the field of XAI, model explanation refers to the compilation of features that allocate importance scores to the contributions made by the provided input data. The goal of this process is to provide a transparent and interpretable explanation of how the model arrives at its predictions by highlighting the significance of different input features. Although the explanation of a model can be produced using the inherent interpretability of the built-in architecture [26,27], such built-in components can change the behavior of the model. By contrast, post hoc explanations can be flexibly applied to conventional DNN models by XAI methods. This approach generally produces explanations by taking the gradient of the prediction function [48]. However, there are several problems that make it difficult to explain models using these methods, namely the complexity of deep learning models, the presence of near- zero gradients for saturated predictions, and the dependence of the sensitivity on the values of the input data. Although approaches to addressing the problems have been proposed [30,33], because these methods utilize the homogeneous nature of target input data such as image pixels, it is hard to apply them to models with heterogeneous data. To explain models with heterogeneous data, there should be no constraints on either the model or the input data. Furthermore, as discussed in Section 2.1, white-box scenarios are not realistic settings. Therefore, black-box explanation methods, which are able to explain any model [28,34,35,36], must be used. Among these, LIME [28] is a model-agnostic method that addresses the problem of model complexity and can explain any data format. Thus, we used LIME as a baseline explanation method.

LIME generates a local explanation model by training a simple model, such as a linear classifier, with neighboring samples close to the input data:

(1) $$\Phi \left(x\right){=}_{g\in G}L\left(f,g,\pi_{x}\right)+O\left(g\right),$$

where *f* is the prediction function, *g* is the approximation function, $\pi_{x}$ denotes neighboring samples around *x*, $L(f,g,\pi_{x})$ measures the difference between *f* and *g* in the space of $\pi_{x}$, and O measures the complexity of the function. The learned parameters of *g* are used to explain the decision of the model function *f* for the input *x* because *g* is the simplified function of *f* in the space of $\pi_{x}$. To obtain a global explanation, LIME uses a submodular pick algorithm that selects a local explanation set without redundant explanations. This algorithm finds the set that achieves the highest coverage of the importance of the features.

## 3. Methods

### 3.1. Malware Classifier Explanations

Dynamic malware analysis involves running a program in a controlled environment and identifying malware by observing its behavior, including system API calls. This technique has demonstrated effectiveness in countering different code obfuscation methods and detecting newly released malware. Past research primarily focused on the API names and neglected the arguments owing to the intricacies associated with the data features. However, Ref. [47] proposed a feature-extraction approach that exploited the hash function and demonstrated outstanding performance relative to the baseline of existing malware classifiers. They further proposed an adaptation of the hash method [49] to encode objects such as arguments.

The feature-selection process in malware classifiers plays a crucial role in determining the effectiveness of machine learning (ML). Given the impracticality of using all features extracted from an entire sample set, many approaches opt to select a reasonable number of features that adequately represent the entire feature set. In prior studies, most classifiers simplified the feature set size by including only those features that appeared in more than a certain number of files. Typically, this threshold is set to 1% of the training set size, and the selection is carried out once retrospectively for the entire dataset. Such mechanisms have specific drawbacks, however; following the same principle, our approach solves the dependency problem by assigning the importance scores of each feature of the instance from the test data distribution. We selected the top-*k* features of each test dataset and used them as votes for the importance measurement of each feature. Hyperparameter *k* can be chosen depending on the size of the features of the data, with the features that receive the most votes compared to the other features considered to be important features.

We used LIME as an explanation method to assign the importance score. However, as previously mentioned, the importance scores assigned by LIME can be dependent on feature sensitivity or scale. Because each feature in heterogeneous data can vary in scale, the importance scores can also be inaccurate. Therefore, instead of directly using the importance scores generated by LIME, we applied the following importance scores to create robust explanations. First, we produced the importance score of each feature using LIME. Second, we selected the top-*k* important features of each test dataset and used them as votes for the important measurement of each feature. Hyperparameter *k* can be chosen depending on the size of the features of the data. The features that receive more votes than others are considered to be important ones.

To enable LIME to interpret heterogeneous data, we exploited the Hamming distance instead of the Euclidean distance to measure the differences between the input data and neighboring samples:

(2) $$\begin{array}{c} L\left(f,g,\pi_{x},k\right)=\frac{1}{n}\sum_{i=0}^{n}\sum_{z,\hat{z}}\pi_{x}\left(z\right){\left(\psi \left(f\left(z\right)\right)\cdot \psi \left(g\left(\hat{z}\right)\right)\right)}_{i}, \end{array}$$

where $g\left(\hat{z}\right)=w_{g}\cdot \hat{z}$ is the class of the linear model, $\pi_{x}\left(z\right)=exp\left(-D{\left(x,z\right)}^{2}/\sigma^{2}\right)$ is the exponential kernel distribution function with width σ, and ψ is the binary Hamming distance between f(z) and $g(\hat{z})$.

Assuming that original model function *f* behaves linearly in the space of $\pi_{x}$, the input data distribution can vary depending on the problem being solved, which might not be accounted for by a fixed sampling method. For a global explanation, LIME utilizes the submodular pick, which selects a set of representative instances and only uses them to extract the feature importance, limiting the coverage of all features. The importance scores used from each feature can depend on the features’ sensitivity, which makes it hard to find a global explanation using a black-box function set.

### 3.2. Mitigating the Influence of Adversarial Examples Using Vadam

The explanation results used to reveal the current malware classifiers rely on specific features that can become targets of AE attackers to reduce their performance. To resolve this problem, we exploited Bayesian optimization to identify an optimal solution for gradient-based optimizers that are not amendable. Because models trained with Bayesian optimization use weight distribution instead of a fixed decision boundary, they robustly apply a wide variety of directions in the feature space [50]. Bayes’ rule can be applied to induce the posterior distribution of the model parameters p(w|D) from the data *D* and prior distribution p(w):

(3) $$\begin{array}{c} p\left(w|D\right)=\frac{p(D|w)p(w)}{p(D)}=\frac{p(D|w)p(w)}{\int p(D|w)p(w)dw} \end{array}$$

However, computing the normalizing term p(D) is intractable, and it is difficult to extend the Bayesian method to large data and large parameters. MCMC methods, Laplace’s method, and variational inference have been proposed to approximate the posterior distribution.

Variational inference (VI) approximates a posterior distribution p(w|D) using a distribution q(w) that can be easily normalized like a Gaussian distribution.

(4) $$\begin{array}{c} L\left(\mu ,\sigma^{2}\right):=\sum_{i=1}^{N}E_{q}\left[\log p\left(D_{i}|w\right)\right]+E_{q}\left[\log\frac{p(w)}{q(w)}\right], \end{array}$$

where μ and $\sigma^{2}$ are optimized by maximizing the variational objective. Recent approaches have made it possible to apply VI to DL by using gradient-based methods. However, the expressiveness of these models is limited.

Natural gradient variational inference (NGVI) improves the complexity of model expressiveness. To obtain natural gradients in the natural parameter space, the Fisher information matrix (FIM) must be calculated.

(5) $$\begin{array}{c} \eta_{t+1}=\eta_{t}+\beta_{t}F{\left(\eta_{t}\right)}^{-1}{▿}_{\eta }L\left(\eta_{t}\right) \end{array}$$

Methods for approximating the FIM have been proposed. The approach in [51] avoids the direct computation of the FIM by introducing minimal representation and expectation parameters. The update rule for the Gaussian mean-field VI is given by:

(6) $$\begin{array}{c} \mu_{t+1}=\mu_{t}+\beta_{t}\sigma_{t+1}^{2}\circ \left[{\hat{▿}}_{\mu }L_{t}\right] \end{array}$$

(7) $$\begin{array}{c} \sigma_{t+1}^{-2}=\sigma_{t}^{-2}-2\beta_{t}\left[{\hat{▿}}_{\sigma^{2}}L_{t}\right] \end{array}$$

Bayesian optimization is used to obtain the output distribution of the new test point marginalized over the posterior distribution of:

(8) $$\begin{array}{c} p(y|x,D)=\int p(y|x,w)p(w|D)dw. \end{array}$$

This Bayesian prediction is then applied to *g* by updating $\psi_{x}\left(z\right)$ with Equation (2) as follows:

(9) $$\begin{array}{c} \pi_{x}\left(z\right)=exp\left(-p\left(y|x,D\right){\left(x,z\right)}^{2}/\sigma^{2}\right). \end{array}$$

#### Example 1: Malware Classification with Neural Networks

**Figure 2:**
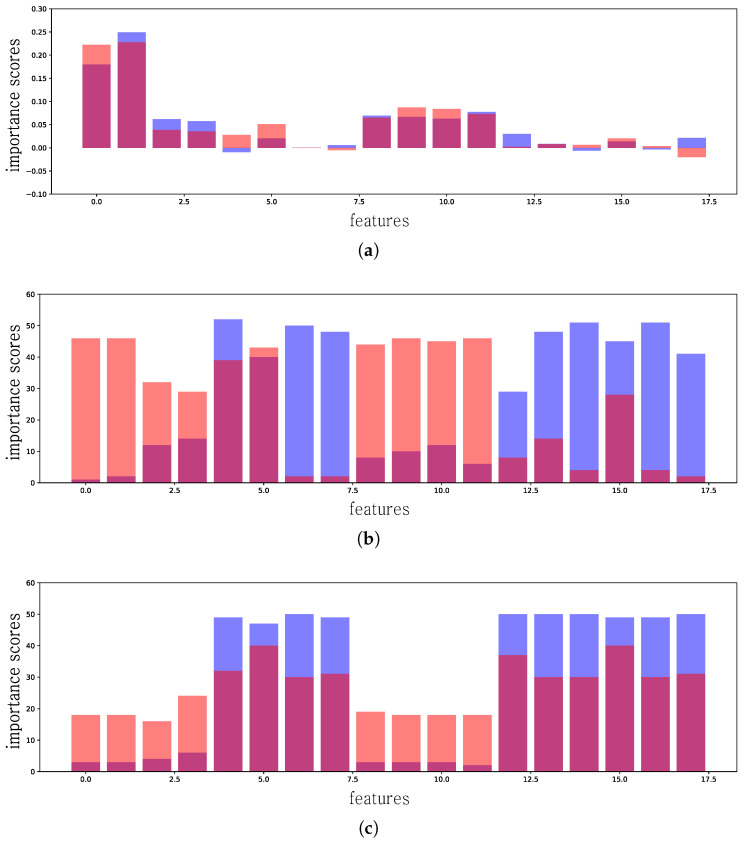
Visualization of PE models obtained by default LIME and our approach. The ground truth test attack distribution is represented by the blue color, while the attack distribution from chosen models is denoted by the pink color. (**a**) PE model results obtained using feature means (default). (**b**) PE model results obtained by Hamming top 10 (ours). (**c**) PE model results obtained by Hamming Vadam top 10 (ours).

For the classification of portable executable (PE) files, the data comprising 18 features are preprocessed by using the hash function [47]. The preprocessed data comprise 1000 consecutive preprocessed API calls, each of which has 102 values; therefore, the data contain 102,000 values. Because importance scores are assigned to all values in the input data, we aggregated the importance scores of 1000 values in each feature and assigned the aggregated score as the importance score of each feature. In Figure 2b, we explain the predictions of a neural network on the PE files to differentiate malicious PE files from the 102-dimensional features. The classifier achieved 95% accuracy, and the score is a major factor of trust in the model. The explanation for this model shows that the prediction of the model still relies on the API name and categories, although API arguments are added as new importance feature factors, which means that removing these arguments will have no effect on this model. From this, it is clear that classifiers can provide false results and cannot be trusted. A detailed explanation of the model is provided in Section 4.7.

## 4. Results

### 4.1. Settings

We ran our experiments on Ubuntu 16.04 (2.6 GHz Intel Xeon E5-2690 v4 CPU and GTX TITAN V (12 GB) GPU). We used the Python programming language (version 3.6.9) and PyTorch library package (version 1.5.1) to implement the DNN, the matplotlib library package (version 3.0.3) to visualize the images, and the LIME library package (version 0.2.0.1) to implement the XAI methods.

### 4.2. Model and Time Complexity

We used a model composed of five convolution layers and one fully connected layer in which the first convolution layer was a 1D layer. As baseline models (random forest, SVM, and bagging models), we used the models of the scikit-learn library package. Because the complexity of the proposed model is higher than that of the linear classifier *g*, which is the approximation function of LIME, the local explanation from *g* can be used to explain our complex model *f*. To generate LIME explanations, we used 500 neighboring samples to explain each point of the input data. The evaluation times of the baseline PE classifier and Vadam PE classifier models were 2 and 40 min per input, respectively. For the PDF classifiers, we used 500 neighboring samples to explain each input data point. The evaluation times of both the baseline PDF classifier and the Vadam PDF classifier models were less than one second per one input.

### 4.3. Classifiers

To implement the SVM, the radial basis function (RBF) kernel with Y=0.0025 and a cost parameter of C=12 was chosen. For the random forest, the number of trees was set to 200, with all other parameters following their default settings in scikit-learn. For the neural network classifier, a 1D convolution featuring one hidden layer was employed as the embedding space. Optimization was carried out using the Adam optimizer [52] with a learning rate of 0.001, a beta rate of 0.5, and a minibatch size of 16.

### 4.4. Datasets

We used two types of datasets, namely malicious PDF and PE files. The dataset containing PDF files was gathered on 20 December 2017 and on 14 March, 19 June, and 17 July 2018, from VirusTotal. This dataset comprises a total of 10,673 files. Additional datasets were sourced from the Contagio dataset, consisting of 9109 benign files and 11,105 malicious files. Furthermore, samples related to Common Vulnerabilities and Exposures (CVE) were obtained from Exploit-db [53], a platform to which proof-of-concept (PoC) codes and files are uploaded. In the experiment, six specific samples from the CVE dataset were used. For PE files, 12 commercial anti-virus engines were used to separate positive and negative samples. Following previous studies [47], we used the same dataset for PE files, in which the data are archived by the data for two months.

### 4.5. Model Evaluation

We used a subset of test examples for LIME without a submodular pick, because the results of a random subset and a subset selected by a submodular pick hardly differ insignificantly. For the global explanation, we used the mean value of importance scores with respect to the class instead of the mean of the absolute value of the scores to compare with our method. The number of test examples for LIME was set to 300 (150 and 150 for each respective class) and 100 (48 and 52 for each class) for the PDF dataset. The neighboring data of each test example were sampled from the quartile group, in which the group statistics were based on the training data. We used a Hamming distance considering the type of dataset as the metric of the distance between neighboring data.

**Figure 3:**
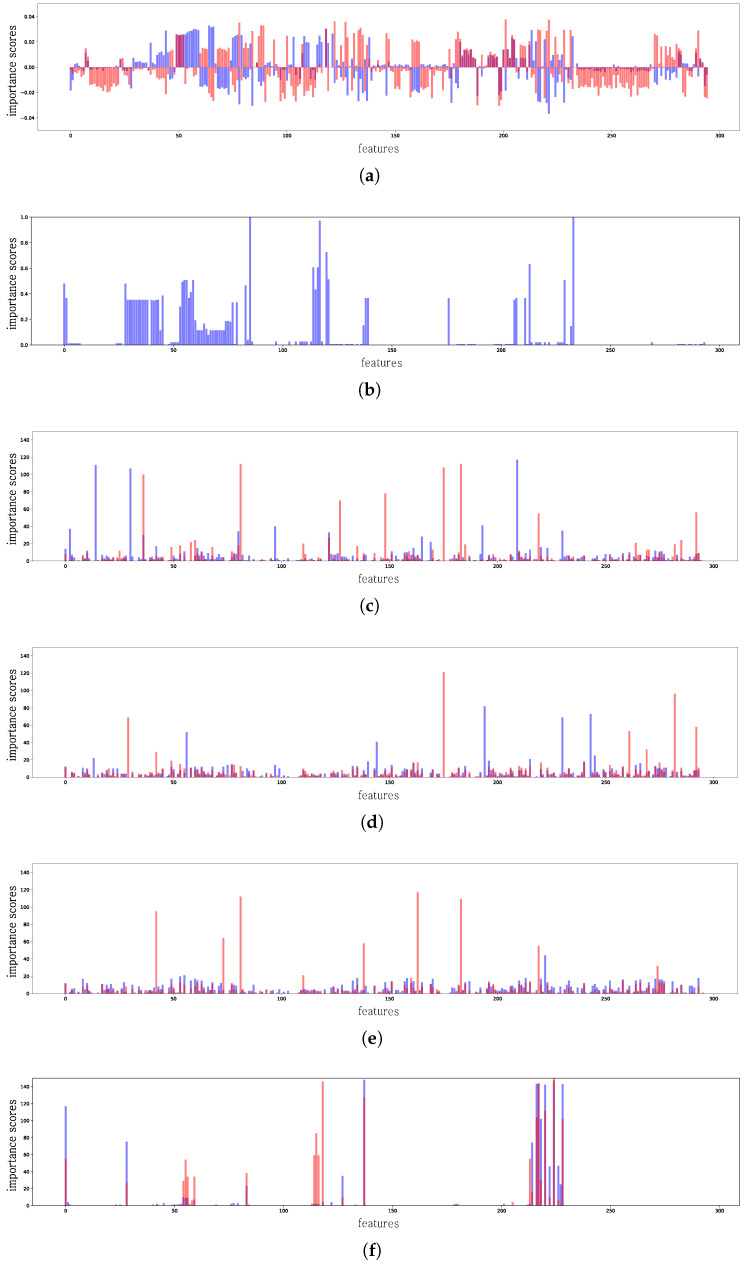
Visualization of representative PDF models: SVM, RF, ensemble, and neural network models, respectively. The ground truth test attack distribution is represented by the blue color, while the attack distribution from chosen models is denoted by the pink color. (**a**) Results for PDF by mean feature (default). (**b**) Ground truth test attack distribution. (**c**) Results for PDF by Hamming top 10 (SVM). (**d**) Results for PDF by Hamming top 10 (RF). (**e**) Result for PDF by Hamming top 10 (ensemble). (**f**) Result for PDF by Hamming Vadam top 10 (neural nets).

LIME [28] extracts the importance of each feature in each test example. The importance scores are represented as a floating number. In a binary classification task, the sign of the importance score indicates the correlated class; for example, if the importance score of a feature is a negative value, it generally implies a positive association with the zero (0) class. Conversely, if the score is positive, the feature is typically positively correlated with the one (1) class. The magnitude of the score’s absolute value denotes the significance, with features having substantial values considered more important than those with smaller values. The results of the global explanation of LIME are denoted as the feature mean, as shown in Figure 2a and Figure 3a. The y-axes of the LIME results represent the importance scores of each feature.

As mentioned in Section 3, our method uses the top-*k* features of each test dataset and uses them as votes for importance scores. We set *k* to 10 in both the PE and PDF datasets and selected the top-*k* features for each dataset according to their absolute importance score values and aggregated votes for all test examples of each class. Therefore, the values of each feature represented the number of votes, with the features with large values being more important than those with small values. The results of the global explanation of our method are denoted as top 10 in Figure 2b. The y-axis of the results of our method represents the aggregation of votes for the top-k importance features in each test dataset.

### 4.6. Results for Dynamic Analyzer

PE files adhere to the most-widely used malware file format in Windows systems, as recognized by AV-TEST in 2017. SecureAge Technology was implemented as the storage to collect the PE files and label them using 12 anti-virus engines. The execution logs were then used to extract features for the PE files. The features encompassed the name, category, and arguments of individual API calls, including elements such file paths or DLLs. Unfortunately, prior studies overlooked the arguments of API calls, leading to the omission of crucial information. However, Ref. [47] extended the Weiger–Berger encoding scheme by adopting the hash method and concatenated features to form a 102-dimensional feature vector, resulting in outstanding performance relative to previous approaches. Table 1 lists the features extracted in this study. The proposed model achieved the best AUC score (98.71%), accuracy (95.33%), and recall (71.48%) on the test datasets. We validated our interpretation model with the dynamic analysis model following the approach of [47], who executed individual test sets in an isolated environment and validated them by collecting runtime behavior. As such results are based on behavioral information, they are known to be much more robust and have a higher detection rate than static analysis [54].

Figure 2a represents the mean feature importance for the 18 features (102 dimensions) obtained by the approximated model via the default LIME, as described in Section 2.3. The x-axis represents the features described in Table 1; specifically, feature 0 indicates *API name*, 1 indicates *API category*, 2 indicates *Integer*, 3 indicates *paths*, 4 indicates *DLLs*, 5 indicates *RegistryKeys*, 6 indicates *URLs*, 7 indicates *IPs*, 8 indicates *numStrings*, 9 indicates *avLength*, 10 indicates *numChars*, 11 indicates *entropy*, 12 indicates *numPaths*, 13 indicates *numDlls*, 14 indicates *numUrls*, 15 indicates *numIPs*, 16 indicates *numRegistryKeys*, and 17 indicates *numMZ*. Features from 2 to 7 are API arguments, and 8 to 17 are string statistics.

Features 8–17 encompass statistical information derived from all the printable strings, which are composed of characters within the range of 0x20 to 0x7f. Of this information, previous studies [43,55] have mainly focused on features 12 to 17. Ref. [47] also focused on statistical information (features from 12 to 17). The approach of Ref. [47] captures the hierarchical information and enabled them to parse numPath (i.e., “C:/a/b/c”), the DLLs registry keys (i.e., “HJEY_”) and IPs (range from 0 to 255 including dots), and numUrls (including substrings to capture domain and organization information separately to contribute more to the feature).

Furthermore, feature 17, composed of strings starting with “MZ”, represents a buffer containing an entire PE file. This aspect is noteworthy as it often introduces vulnerabilities [56]. From the information above, we can expect that a good interpretation model should strongly emphasize features between 12 and 17.

According to the default LIME interpretation (Figure 2a), the model only uses a positive class to classify its maliciousness. Unfortunately, the interpretation is inconsistent with interpretations based on previous studies for the models with malicious PE features. Previously developed malware classifiers were trained without API arguments, which indicates that feature dimensions between 0 and 12 only classify their maliciousness. For example, features between 0 and 12 contain information such as:

$$\begin{array}{c} \mathrm{basename}:\mathrm{lsasrv}.\mathrm{dll}\\ \mathrm{imgsize}:45056\\ \mathrm{baseaddr}:\text{0x7fefcc70000} \end{array}$$

If API arguments are used for the trained model, the model should highlight the features between 8 and 13 (dimensions between 85 and 102) because most exploits are detected by filtering the URLs, IPs, and statistics of API arguments. Figure 2b represents the feature importance score calculated by the top-10 features described in Section 3. Our approach of measuring feature importance clearly highlights the features between 8 and 13, which all malware models consider as importance features for the classification. Figure 2c shows the interpretation results obtained by the model (Section 3.2), which adopts Vadam to perform variational inference for the PE classifier. From Figure 2c, we can observe that the importance features between 12 and 17 have stronger highlights and less-important features such as API names and categories than those shown in Figure 2b. The results indicate that the posterior distribution obtained from Bayes’ rule helps to estimate the uncertainty of the models. We validated our interpretation model using the dynamic-based model and were able to interpret a trained model based on heterogeneous datasets.

### 4.7. Results for Static Analyzer

Table 2 lists the performance results obtained by the representative PDF classifiers described in Section 2.2. To validate the visualization performance, we deliberately removed the subtree of PDF structures, which suggests that deliberately removed subtrees should be ignored when they are visualized using a model that is approximated by LIME. Seventy-five percent of the subtrees were randomly removed (replaced by the value −1) to validate our assumptions such as:

$$\begin{array}{cc} \; & /\mathrm{Root}/\mathrm{Pages}/\mathrm{Kids}/\mathrm{Annots}/\mathrm{Rect}\\ \; & /\mathrm{Root}/\mathrm{AcroForm}/\mathrm{Fields}/\mathrm{Kids}/\mathrm{Kids}/\mathrm{Rec}\\ \; & /\mathrm{Root}/\mathrm{Pages}/\mathrm{Kids}/\mathrm{Contents}/\mathrm{Filter} \end{array}$$

Figure 3a illustrates the mean feature importance for the 295 PDF features in the model approximated by the default LIME, as described in Section 2.3. According to the default LIME interpretation, the model cannot interpret deliberately removed subtrees. From the experimental results, all features were highlighted with forcibly limited subtrees, which indicates that the default LIME is not useful for interpreting PDF datasets.

Figure 3a shows a comparison of the ground truth test attack distribution results obtained by the proposed model and the representative PDF classifiers listed in Table 2. In previous studies, machine-learning-based PDF classifiers were often easily evaded by the AE through the manipulation of node structures. The most-common attack simply repeats feature manipulation based on genetic programming until an evasion succeeds.

Our interpretation model revealed that none of the ML-based models followed the ground truth test attack distribution. Although less-important features with lower importance mean scores were highlighted, none of the ML models failed to take into account removed subtrees. However, it is seen from Figure 3f, which shows the performance of the NN-based variational model (Section 3.2) for the PDF classifier, that the visualization from our interpretation approach clearly ignores the deliberately removed features. Among the 256 PDF features, only partial features (25%) were seen to have meaningful feature values, and most of the feature values are marked as −1 to represent the sparse information of the tree structure. Inspecting the x-axis, the result clearly shows that more than 50% of the features are not represented, and the mean score of the data value is clearly different from those of other ML-based classifiers.

## 5. Discussion

Deep learning (DL) has proven effective in representing various types of high-dimensional data in a low-dimensional Euclidean space. In the context of high-dimensional data, there is a common challenge in which information can be lost during the feature-extraction process. For instance, in many modern malware detectors, only a subset of features is chosen from a larger pool for training, and the feature-selection process significantly influences the detection performance. The feature-selection process is pivotal in determining the effectiveness of ML. The use of all features from an entire dataset is impractical because this will create high sparsity and high dimensionality. To address this, various studies have proposed unique approaches to enhancing feature selection. For example, a feature can be scored in terms of frequency or based on divergence, and a threshold or desired number of features in the set can be set for filtering. However, these approaches encounter challenges in achieving a balanced inclusion of extracted features from both sides (benign vs. malicious), as malicious files often contain distinctive features. When assessing machine learning classifiers for security applications, relying solely on accuracy and false positive rates is inadequate. Existing metrics used in machine learning theory do not sufficiently measure the robustness of classifiers against AEs.

From our proposed explanations, we noticed that some of the features not covered during the training phase provide a space for AEs. This occurs if the feature distribution is not well designed, so that all features are not covered during the feature-extraction phase. Hence, mitigating the impact of manual processing can enhance the performance of detectors trained on high-dimensional data. We propose that successful representation and abstraction of all information without the need for manual processing during the training of DL models could lead to a substantial enhancement in the evasion performance of malware classifiers. The introduction of Bayesian optimization allows models to utilize robust feature spaces via an unbiased manifold representation. In conjunction with conventional security measures such as accuracy, our post hoc explanation methods will help to verify malware classifiers. Our methods expand the application of XAI methods to robust explanations of models for heterogeneous data. The best-performing models often increase defense accuracy using gradient masking, which provides a false model gradient [57]. Whether intentional or inadvertent, gradient masking can occur when loss functions distort the loss surface in the data space. A malware classifier must demonstrate practical effectiveness by proving its security usefulness using various metrics. In this respect, we believe that our work will provide guidance on the transferability of malware classifiers and contribute to the prevention of a false sense of security in malware classifiers.

## 6. Conclusions

We analyzed the transparency of existing explanation methods in the context of malware classification, finding that the individual representative explanation models often struggle to discern crucial features within heterogeneous data. This limitation arises from the inability of the submodular pick in LIME to effectively interpret such diverse data. To address this issue, we introduced an approach that selects the top-k importance features, enabling a more-robust application of the explanation method to heterogeneous data. Consequently, our approach yielded improved interpretation results closely aligned with the ground truth labels. Additionally, we observed that most deep-learning-based malware classification models such as AEs exhibit vulnerability to bounded attacks. By replacing the optimizer with Bayesian optimization, we obtained an enhancement in robustness against AEs.

## Figures and Tables

**Table 1:** PE feature representations.

Feature Name	Types	Dimension
API name	Strings	8
API category	Strings	4
API arguments		
Integer	Integers	16
Paths	Strings	16
DLLs	Strings	8
RegistryKeys	Strings	12
URLs	Strings	16
IPs	Strings	12
String stats	Strings	10

**Table 2:** Performance results of representative DL-based PDF malware classifiers.

Classifiers	Accuracy	AUC
SVM (Hidost 13’)	96.46%	0.9886
RF (Hidost 16’)	96.45%	0.9880
Ensemble (PDFrate-v2)	99.37%	0.9932
Neural nets (PE-based)	99.93%	0.9999

## Data Availability

Data are contained within the article.
